# Substitution of Hospital Care with Primary Care: Defining the Conditions of Primary Care Plus

**DOI:** 10.5334/ijic.2446

**Published:** 2016-04-14

**Authors:** Sofie Johanna Maria van Hoof, Mariëlle Elisabeth Aafje Lydia Kroese, Marieke Dingena Spreeuwenberg, Arianne Mathilda Josephus Elissen, Ronald Johan Meerlo, Monique Margaretha Henriëtte Hanraets, Dirk Ruwaard

**Affiliations:** Department of Health Services Research, Faculty of Health, Medicine and Life Sciences, Maastricht University, Maastricht, The Netherlands; Department of Health Services Research, Faculty of Health, Medicine and Life Sciences, Maastricht University, Maastricht, The Netherlands; Centre for Technology in Care, Zuyd University of Applied Sciences, Heerlen, The Netherlands; Primary Care Organisation Care in Development (ZIO), Maastricht, The Netherlands; Department of Patient and Care, Academic Hospital Maastricht (azM), Maastricht, The Netherlands

**Keywords:** Substitution, primary care, hospital care, preconditions

## Abstract

**Objective:**

To analyse barriers and facilitators in substituting hospital care with primary care to define preconditions for successful implementation.

**Methods:**

A descriptive feasibility study was performed to collect information on the feasibility of substituting hospital care with primary care. General practitioners were able to refer patients, about whom they had doubts regarding diagnosis, treatment and/or the need to refer to hospital care, to medical specialists who performed low-complex consultations at general practitioner practices. Qualitative data were collected through interviews with general practitioners and medical specialists, focus groups and notes from meetings in the Netherlands between April 2013 and January 2014. Data were analysed using a conventional content analysis which resulted in categorised barriers, facilitators and policy adjustments, after which preconditions were formulated.

**Results:**

The most important preconditions were make arrangements on governmental level, arrange a collective integrated IT-system, determine the appropriate profile for medical specialists, design a referral protocol for eligible patients, arrange deliberation possibilities for general practitioners and medical specialists and formulate a diagnostic protocol.

**Conclusions:**

The barriers, facilitators and formulated preconditions provided relevant input to change the design of substituting hospital care with primary care.

## Introduction

International comparative analysis has shown that healthcare costs per capita have increased at a faster pace in the Netherlands than elsewhere in Europe during the last decade [1]. If healthcare expenditures continue to rise at this pace in the Netherlands, it is expected that costs of care, as a percentage of the Gross Domestic Product, will rise from 13% in 2010 to 22–31% in 2040 [2]. This growth rate threatens future accessibility and affordability, and hence sustainability of the Dutch healthcare system [3].

It is therefore not surprising that redesigning the healthcare system to decrease rising costs is high on the Dutch political agenda. In 2012, the ‘Dutch Taskforce Healthcare Expenditures’ formulated recommendations to slow down rising costs: (a) care should ‘go back to the basics’, with the general practitioner still as gatekeeper; (b) care should be provided at the ‘right place’, with more task substitution to primary care; and (c) all parties should share a mutual responsibility for controlling healthcare costs [3]. Similar recommendations were made in the 2012 ‘Agenda for Health care’ developed by 14 Dutch care organisations, health insurance companies, and provider and patient associations [4]. More recently, the Dutch Ministry of Health, Welfare and Sport, healthcare organisations, health insurance companies and patient organisations have agreed that the volume growth for hospital care should be limited to 1.5% in 2014 and 1% per year from 2015 until 2017. In contrast, primary care is allowed to grow by 1% in 2014 and 1.5% per year from 2015 until 2017 to stimulate task substitution from hospital care to primary care and to avoid unnecessary referrals to hospital care [5].

As recommended by health insurance companies, the Dutch Ministry of Health, Welfare and Sport has labelled nine regions across the Netherlands as pioneer sites in April 2013. These regions started experiments to avoid unnecessary expensive care and to achieve improvement on the quality of health care. The minister will actively monitor these regions over the coming years until 2017. One of these regions is Maastricht-Heuvelland with initiative ‘Blue Care’ [6]. Blue Care aims to achieve more sustainable care by initiating different interventions according to the Triple Aim principle [7]. They state that to achieve high quality care improvements, three aims should be linked: reduced care costs per capita should coincide with improved population health and patient experiences [7,8,9].

In this perspective, shifting tasks from hospital care to primary care is proposed as one option to comply with these principles, and may lead to advantages in terms of quality of care, fewer referrals to hospital care, shorter waiting lists and lower costs for patients [10,11,12,13,14,15,16,17]. Therefore, as part of the Blue Care initiative, a so called Primary Care Plus intervention was developed with the aim of creating substitution and stimulating integrated care by allowing medical specialists to perform consultations within primary care.

Because barriers and facilitators of Primary Care Plus have not systematically been investigated yet, a feasibility study of Primary Care Plus was conducted in a small setting within the Blue Care initiative. Understanding the barriers and facilitators for implementation is firstly crucial to assess the feasibility of Primary Care Plus, and to find out what preconditions are required for a successful roll-out of Primary Care Plus in the entire Blue Care initiative. An evaluation of a quality process contributes to gaining insight into the feasibility and experiences of stakeholders with a specific intervention in order to develop and improve certain quality improvement interventions [18]. Therefore, our research questions were *(1) What are the barriers and facilitators of Primary Care Plus? and (2) What are the necessary preconditions to successfully implement Primary Care Plus in the Blue Care initiative and beyond?* These preconditions can provide input for other regions that will start implementing Primary Care Plus and input for policy implications at the national level. The effects of Primary Care Plus on the Triple Aim principle will be subject of future studies.

## Methods

### Design

This study was part of a mixed methods study on the feasibility of the Primary Care Plus intervention which was conducted at the pioneer site Blue Care. In this descriptive study, we applied a qualitative design. Data regarding process information was collected through interviews with involved general practitioners and medical specialists and through notes from all meetings with various working groups to identify barriers and facilitators and proposed solutions in order to formulate preconditions for Primary Care Plus.

### Setting

The Blue Care initiative is a partnership between the only primary care organisation in the region ‘Care in Develop-ment’ (in Dutch ‘Zorg In Ontwikkeling’), the academic hospital Maastricht, the patient representative foundation ‘House of Care’ (in Dutch ‘Huis voor de Zorg’), and the most dominant health insurance company VGZ in this region. The name Blue Care is used as an analogy for green power to indicate the importance of behavioural change to achieve sustainable care for the future. Blue Care works according to pillars which are defined in a covenant [6]. The first pillar defines the need for changing the healthcare system and incorporates interventions in the care process. One of these interventions is Primary Care Plus. The last pillar concerns the need for achieving behavioural change in citizens, patients, health professionals, health managers and financiers. The partnership between Care in Development and the academic hospital Maastricht is already known for its long-term relationship and collaboration history, for example in the ‘joint consultation model’ [16,17,19].

### Intervention

The seven-month Primary Care Plus feasibility study ran from April 2013 till January 2014 (July and August 2013 were excluded) as part of the Blue Care initiative. Seventeen general practitioners from various practices were able to refer non-acute patients, about whom they had doubts regarding diagnosis and/or treatment and whether or not to refer the patient to the hospital to Primary Care Plus. Medical specialists performed Primary Care Plus in consultation rooms at general practitioner practices. The medical specialists examined the patients in a shorter time compared to a consultation in the hospital, and provided advice to the general practitioner afterwards. The general practitioner remained responsible for the patient. The maximum number of visits to Primary Care Plus per patient and per medical complaint was two and the maximum consultation time was 20–30 minutes for a first consultation and 10–15 minutes for a recurrent consultation, depending on the medical specialty. Medical specialists only had access to (diagnostic) materials which were available in the general practitioner practices and thus were only able to perform care not requiring the facilities of the hospital.

The involved general practitioners consisted of two groups:

The intervention group consisted of ten general practitioners working in six practices with 17,416 patients and 13 medical specialists representing five medical specialties (internal medicine (*n* = 2), cardiology (*n* = 1), neurology (*n* = 1), dermatology (*n* = 2) and orthopaedics (*n* = 7) from the academic hospital Maastricht who performed consultations on a weekly or two-weekly basis within their general practitioner practices.

The referral group consisted of seven general practitioners working in six practices with 14,906 patients. In these practices the medical specialists were not represented. Patients eligible for Primary Care Plus were referred to one of the six practices of the intervention general practitioners.

General practitioners in Maastricht-Heuvelland work with referral organisation TIPP (Transmural Interactive Patient Platform). The call centre of TIPP guides patients referred to specialist care by their general practitioner for an appointment. TIPP collected information about quality of care and waiting lists for healthcare organisations in the region.

## Measurements

### Interviews with general practitioners and medical specialists

Prior to the start of the feasibility study, semi-structured face-to-face interviews with involved general practitioners (*n* = 4), involved medical specialists (*n* = 4) and chiefs of medical departments at the academic hospital Maastricht (*n* = 3) were conducted by researchers (M.S. and A.E.). Interviews took place at the workplaces of the interviewees. They were recorded using audio-recording devices and lasted on average 37 minutes. Topics included: (1) expectations of stakeholders regarding Primary Care Plus; (2) definition of Primary Care Plus; (3) expected outcomes of Primary Care Plus; (4) practical implications of Primary Care Plus; and (5) the most important indicators for measuring the outcomes of Primary Care Plus. Recording files were transcribed and coded manually according to barriers, facilitators and policy adjustments.

### Observational notes: meetings of working groups

**Figure 1:**
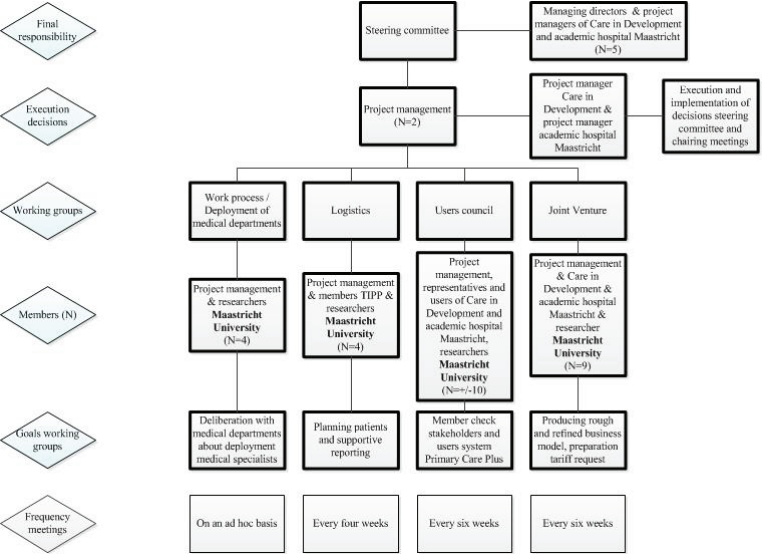
Organisation chart of the Primary Care Plus project.

To manage the Primary Care Plus project, different working groups, which were hierarchically positioned under the steering committee and project management, were composed (see Figure 1). The working groups focused on the deployment and formation of specialists from medical departments, logistics, monitoring the practice of Primary Care Plus in a users’ council, and creating a business model in a joint venture, respectively. The steering committee was responsible for making decisions at the managerial level and had the final responsibility. The project management took care of the implementation of these decisions and was member of each working group. The working group for deployment of medical departments discussed the employability of the medical specialists per department. The logistics working group monitored the organisational process and took care of the IT and logistical issues. Usability and feasibility of the Primary Care Plus process were checked by the users’ council. The joint venture working group was concerned with organising a business model and arranging necessary legal requirements. In addition to this organogram, a research team was created to make sure the process could be studied scientifically. The meetings of the project management and all working groups (*n* = 45) were observed (S.H.) and documented by note keeping and also coded according to barriers, facilitators and policy adjustments.

### Focus group with involved Primary Care Plus stakeholders

During the study, intermediate results about barriers and facilitators were presented to involved general practitioners (*n* = 8) and medical specialists (*n* = 8) during two focus groups. The aim of these focus groups was receiving feedback from stakeholders about their experiences with these barriers and facilitators and about possible policy adjustments. These focus groups were audio recorded and lasted on average 79 minutes.

## Analysis

The interviews and the notes from the meetings of the project management and working groups were analysed using a conventional content analysis method [20] resulting in barriers and facilitators. The barriers and facilitators were then combined by S.H., M.S., M.K. and D.R. in seven main themes which are based on the issues that were most frequently mentioned by interviewees, participants of the project management and working groups and participants of the focus groups. The barriers and facilitators resulted in policy adjustments during the feasibility study in Blue Care. General preconditions were formulated based on these barriers, facilitators and policy adjustments. The analysis process and the formulation of general preconditions was carried out by S.H. and regularly discussed and updated with M.S., M.K. and D.R.

### Member check with the project management

A member check, also known as respondent validation, is a way to discuss the results found in the interviews and the observational notes [21]. To perform a member check and to analyse whether the project managers of Care in Development and the academic hospital Maastricht recognised the barriers, facilitators and policy adjustments and pre-conditions based on the results of this study, a meeting was organised.

This Primary Care Plus feasibility study was exempt from review by the Medical Research and Ethics Committee (Application number: 13-5-042) since it was not liable according to the Dutch Medical Research (Human Subjects) Act [22].

## Results

The interviews, the notes from meetings of the project management and working groups and the focus groups resulted in barriers and facilitators which were described in the second column in Table 1. These barriers and facilitators were categorised into seven main themes formulated by S.H., M.S., M.K. and D.R., described in the first column. The third column depicts the policy adjustments the Blue Care steering committee opted to change and improve Primary Care Plus in response to the barriers and facilitators found. In response to these barriers, facilitators and policy adjustments, general preconditions were formulated which initiators should keep in mind when starting Primary Care Plus.

**Table 1:** Overview of main themes, barriers and facilitators, policy adjustments in Blue Care and preconditions of Primary Care Plus.

Main theme	Barriers (b) and facilitators (f)	Policy adjustments in Blue Care	Preconditions

I Arrangements	Signed covenant Blue Care-shows mutual trust between all stakeholders (f) Potential lack of long-term commitment (b) Willingness of stakeholders to redesign structure of health care in region (f)	Creation of a business model Arrangement of a special tariff for Primary Care Plus Organisation of legal agreements with Dutch Care Authority	– Put effort in mutual trust between stakeholders – Designate an integrator – Pursue a common goal (substitution) – Arrange legal agreements
II IT-systems	Referral organisation TIPP was not able to make appointments in hospital information system (b) Appointment application of referral organisation TIPP was not available in general practitioner practices (b) Hospital and general practitioner information systems were not linked with each other (b)	Possibility for referral organisation to make appointments directly in the agenda system of the medical specialties +3. Creation of a Primary Care Plus application in which all stakeholders (general practitioners, medical specialists, TIPP) have access	– Arrange a collective IT-system where appointment system and information systems from general practitioners and the (academic) hospital are integrated
III Participation and involvement of all care providers	Assistants of general practitioners were not informed about Primary Care Plus and did not know how to support medical specialists working in their practice (b)	Organisation of information sessions for all care providers Development of an internet forum, where all information about the project is available	– Make sure all stakeholders are informed about the ins and outs of the intervention and their responsibilities, e.g. through information sessions
IV Profile of medical specialist	General practitioners only accepted advice from medical specialists who had considerable experience in their working field (b/f) Medical specialists had different coping styles with Primary Care Plus and the paradigm shift in health care (b/f)	+ 2. Determination of a profile for the medical specialist in Primary Care Plus: Senior Generalist Medical specialist should share the conviction of the necessity of substitution	– Qualifications for an appropriate profile of an eligible medical specialist who will be working in Primary Care Plus
V Referral pattern	The adherence area of this feasibility study was too small resulting in too few referrals for efficient consultation time in Primary Care Plus (b) General practitioners from referral practices feared referring their patients to intervention practices (b) General practitioners from intervention practices experienced a relatively low threshold when referring to Primary Care Plus (b/f) General practitioners experienced difficulties in whether or not to refer a patient to the hospital or Primary Care Plus (b)	1. +2. +3. Opening independent Primary Care Plus centre(s) 4. Various proposed solutions: Medical specialists giving feedback to general practitioners about the referrals Discussion in multidisciplinary teams about the referrals Referral according to the default principle^a^ Referral according to the International Classification of Primary Care coding system	– Make sure that consultation time will be efficiently planned (e.g. by centralising Primary Care Plus) – Make sure that general practitioners are able to deliberate with medical specialists about referral uncertainties
VI Communication between general practitioner and medical specialist	Advice letter from medical specialist arrived too late at general practitioners office (b) ‘Out of sight, out of mind’ – fewer referrals from referral practices compared to intervention practices (b) Deliberation was experienced as very valuable and crucial to continue Primary Care Plus (f)	Track and trace system/application +3. Various opportunities to deliberate: E-mail, telephone, walk-in consultation hours for general practitioners (and their patients), video conference and joint consultations	– Arrange various possibilities for general practitioners to deliberate with medical specialists
VII Arrangements regarding diagnostic procedures	Uncertainty about the responsibility for requesting diagnostics (b) Different diagnostic facilities for specialists available in general practitioner practices (b) Not enough diagnostic tools available in Primary Care Plus depending on the medical specialty (b) No access to diagnostic results if diagnostic tests were being performed in organisations other than the academic hospital (b)	2. Opening Primary Care Plus centres where diagnostic tools are available	– Create a diagnostics protocol, in which responsibilities are defined – The cooperating laboratory/organisation where diagnostics are performed should be defined – Consider which medical specialties are eligible for Primary Care Plus – To avoid double diagnostic requests, medical specialists should have access to previous diagnostic results, also from organisations other than the hospital

^a^Default principle: All patients who need a referral are eligible for Primary Care Plus unless they need acute care or the facilities of the hospital.

The analysis showed that participants in this study came up with similar barriers, facilitators and topics. In addition, the member check with the project management showed that they agreed with the results and the categorisation into main themes.

### Arrangements

Based on the interviews and notes from the working group meetings, that all stakeholders underlined the pillars of Blue Care and Primary Care Plus seemed an important step to secure the future of Primary Care Plus [6]. Covenant Blue Care was signed by the boards of all stakeholder organisations which showed strong mutual trust. All parties acknowledged the need for collaboration and showed their willingness for blurring the boundaries between primary and hospital care. Interviews made clear that all general practitioners, medical specialists and project leaders involved, broadly praised the willingness of all stakeholders to collaborate in this project, even if market positions were threatened. They believed Primary Care Plus has the potential to substitute hospital care with primary care. However, to keep all stakeholders committed in the future, there was an increasing demand for the creation of a business model, a special tariff for Primary Care Plus consultations and arrangements in legal terms (e.g. adapting the Competition Act) (see Table 1 ‘I’). The business model mentioned includes a description of the possibilities of Primary Care Plus, the target population and a broad cost-benefit analysis. Because Primary Care Plus is assumed to be cheaper compared to hospital care, Blue Care is willing to negotiate a cheaper tariff for Primary Care Plus consultations with health insurance companies. In addition, the steering committee is also arranging to indemnify patients for paying mandatory deductibles for a consultation in Primary Care Plus. However, a prerequisite for this negotiation is that Blue Care arranges legal agreements with the health authorities in the Netherlands according to The Dutch Law on Competition [23] to approve possible cartel formation. These considerations are also preconditions for other Primary Care Plus initiatives in this country and beyond.

### IT-systems

During the feasibility study, general practitioners and medical specialists experienced problems regarding the non-existing link between these systems (Table 1 ‘II’). General practitioners and medical specialists have their own information systems, and TIPP has its own TIPP-application. The steering committee will take care of the creation of a Primary Care Plus application where all medical systems are linked and accessible via the portal of the application. A general precondition on IT-systems is that stakeholders should be able to have access to all different IT-systems involved.

### Participation and involvement of all care providers

Members of the working groups experienced great involvement by all participating general practitioners, medical specialists and project leaders of Primary Care Plus. However, not all employees of general practitioner practices (e.g. assistants) were involved and informed about the ins and outs of the project (see Table 1 ‘III’). This sometimes resulted in unfamiliarity with medical specialists working in their general practitioner practice, and in unawareness about how to act when patients did not show up for an appointment. As a solution, the steering committee organised information sessions for care providers and gave the assignment to build an internet forum where information about the project is placed. A precondition to start with Primary Care Plus is that all parties involved should be aware of the ins and outs of the intervention and their responsibilities and be kept informed about these responsibilities.

### Profile of medical specialist

Some general practitioners mentioned their concern in interviews and meetings of the users’ council about the profile of the medical specialist working in Primary Care Plus (Table 1 ‘IV’). Those general practitioners rather took advice from experienced medical specialists than from unexperienced specialists. Others were particularly positive about a junior medical specialist in their practice because he was willing to work according to the substitution model, and put a lot of effort in the project. All stakeholders agreed that the attitude of medical specialists should be in line with the substitution model following a generalised approach to assure that he/she does not use Primary Care Plus as a certain referral station for hospital care. In a hospital, specialists work according to the principle of people being ill until proven otherwise, while in Primary Care Plus they should work according to the belief of people being healthy until proven otherwise. This required a shift in thinking and a certain level of experience. The steering committee formulated a profile for medical specialists working in Primary Care Plus based on a generalist approach and seniority with several years of experience. A precondition is to create qualifications for an appropriate profile of an eligible medical specialist in Primary Care Plus.

### Referral pattern

At the organisational level, an important issue threatening the introduction of Primary Care Plus was related to the inefficient and limited use of consultation hours due to the small scale (see Table 1 ‘V’), leading to resistance, especially from dermatologists and orthopaedics. They mentioned not continuing the project if a medical specialist had to come to a general practitioner practice for only one patient. Notes from the users’ council revealed that participating referral general practitioners did not use Primary Care Plus although they acknowledged the advantages, because they were afraid of losing patients when referring to a medical specialist in another general practitioner practice. Another threat was the relatively low threshold for intervention general practitioners to refer to Primary Care Plus. General practitioners stated that working together within a general practitioner practice resulted in good working relations between general practitioners and medical specialists leading initially to more referrals; patients about whom the general practitioner felt insecure were now referred to the medical specialist instead of treated by the general practitioner him/herself. To fill consultation time efficiently and to avoid the overuse of care due to close working relations, the steering committee came up with the concept of independent Primary Care Plus centres. Here, medical specialists would work in a neutral environment without general practitioners in their direct surrounding. Although some general practitioners were positive about this decision, other general practitioners mentioned they were afraid of losing their close contact between general practitioners and medical specialists and hence knowledge transfer.

Another issue is the difficulty and uncertainty general practitioners experienced in referring eligible patients to Primary Care Plus. As a result, medical specialists sometimes saw patients in Primary Care Plus who should have been referred directly to hospital care or who could have been treated by the general practitioner him/herself. Proposed solutions of the steering committee were creating possibilities for general practitioners to deliberate with medical specialists about referral uncertainties, referring according to the default principle (all referrals to Primary Care Plus, unless they need acute care and the facilities of the hospital), and using the International Classification for Primary Care coding system for selecting eligible patients. In the last option, ICPC codes of non-complex complaints and symptoms are identified, and when a general practitioner enters such a code in his/her information system, this referral will automatically be converted to a Primary Care Plus referral.

In addition, cardiologists reported that too few patients were eligible for consultations in Primary Care Plus because the majority of the cardiology patients entered the care system with acute problems. Therefore, the department of cardiology is considering an alternative appearance of Primary Care Plus in which stable chronic cardiac patients will have control consultations in Primary Care Plus instead of in the hospital. Preconditions to facilitate referrals are creating possibilities to deliberate between general practitioners and medical specialists and the efficient use of consultation time in order to give Primary Care Plus the right to exist.

### Communication between general practitioner and medical specialist

Intervention general practitioners were particularly positive about having the medical specialist in their direct surrounding creating opportunities to deliberate about patients and uncertainties (see Table 1 ‘VI’). However, in the users’ council, general practitioners noted some communication problems. Medical specialists had to send their advice letter meant for the general practitioner first to their hospital information system before releasing the letter to the general practitioner, resulting in delayed advice for general practitioners and uncertainty about whether follow-up action was expected. As a solution, the steering committee decided that medical specialists should dispatch the advice letter to the general practitioner before sending the medical information to their hospital information system. The Primary Care Plus steering committee wanted to maintain the positive side of deliberating with medical specialists organising ‘joint consultation hours’ in the Primary Care Plus centres. Joint consultation is a consultation where several general practitioners are able to examine and discuss their patients with a medical specialist at the same time [19]. Another possibility is to make telephone appointments with medical specialists. A general precondition for implementing Primary Care Plus is to arrange at least one possibility, but preferably more possibilities, for general practitioners to deliberate with medical specialists.

### Arrangements regarding diagnostic procedures

The last theme regarded the arrangements for diagnostic procedures (see Table 1 ‘VII’). The premise of the Primary Care Plus project was that general practitioners would remain medically responsible for the patient. However, general practitioners and medical specialists believed that they both should be able to make diagnostic requests, depending on who would discuss the test results with the patient. In addition, specialists experienced variability in the availability and the type of facilities in general practitioner practices. Some general practitioners had various diagnostic tools in their practice (e.g. needles, urine jars, instruments to take biopsies) but were reluctant to make them available to medical specialists. Cardiologists and orthopaedics experienced a deficiency of available diagnostic tools in Primary Care Plus. ECG devices were lacking for cardiology and orthopaedics needed an X-ray to be able to diagnose patients. Neurologists and internists mentioned that they requested fewer diagnostic tests in Primary Care Plus compared with usual hospital care.

Furthermore, general practitioners had different diagnostic collaboration partners for performing diagnostic tests, and medical specialists only had access to the results of tests performed in the academic hospital. This resulted in a discussion between general practitioners and medical specialists about where to perform diagnostic tests. To solve these disagreements and insecurities regarding diagnostic procedures, the Primary Care Plus consulting rooms in the Primary Care Plus centre(s) will be decorated and facilitated by medical specialty. However, the uncertainties regarding responsibilities for requesting diagnostic tests and the co-operating diagnostic laboratories remained. As a precondition a diagnostic protocol should be defined.

## Discussion

### Main findings

This study uncovered a number of issues regarding the implementation of Primary Care Plus. For some barriers, policy adjustments were made, nevertheless some barriers remained. To successfully implement a quality improvement intervention on the Triple Aim parameters, there is a need for an initiator and promoter who inspire others and a need to obtain top-down approval by the boards of involved organisations [7]. Mutual trust is essential for achieving that. The Blue Care region is characterised by a long history of collaboration between primary and hospital care which resulted in several healthcare innovations [24,25]. Care in Development and the academic hospital Maastricht acknowledged their integrators role. They felt social responsibility for effective deployment of available health resources according to the Triple Aim principle and therefore designed the covenant Blue Care and negotiated with health insurance companies about a special tariff for Primary Care Plus. A tariff for Primary Care Plus, lower than for hospital care, seems reasonable. However, to accomplish ‘real substitution’, the (academic) hospital (Maastricht) should perform care in Primary Care Plus without compensating the potential volume loss and consequently decreasing incomes. However, dedication to the Blue Care-thought is not enough. If Primary Care Plus constitutes substitution, a decline in the number of patients referred to the hospital will be the result. As a solution, health insurance companies should come to an agreement with the academic hospital to alleviate the decline in revenues. Such an agreement is lacking at the moment, making the long-term commitment of the academic hospital Maastricht to the Primary Care Plus project breakable. Furthermore, literature shows that successful integrated care could only be accomplished by sharing financial structures between organisations which try to achieve integrated care [26]. The agreement mentioned above could therefore be an intermediate solution in overcoming fragmented financial structures and in reducing the threat of not accomplishing successful integrated care in Blue Care.

### Reflection with existing literature

Working in cooperation projects requires a representative formation of stakeholders who share the personal belief in project goals [27]. This means that particularly the shared conviction of substitution is an important precondition in the profile of medical specialists working in Primary Care Plus. The dedication to a Primary Care Plus intervention and having the conviction of substitution may be an issue in the case of medical specialists working in general partnerships and revenues decline due to substitution. For them, this could have a major impact because they work according to the principle of fee for service while medical specialists in academic hospitals earn a fixed salary.

A crucial precondition for the success of Primary Care Plus is the efficient use of consultation hours and time of medical specialists. A system that works properly with efficient planning contributes to and supports an effective system of primary health care [28]. In other European countries, outreach clinics have been a topic for research. The proposed model in which specialists conduct Primary Care Plus consultations in independent Primary Care Plus centres closely resembles this care form. Literature has shown that without efficient planning and efficient use of consultation hours, outreach clinics could not be cost effective [11,29,30]. It is therefore essential to have an adherence area that provides enough patients for efficient use of consultation hours and thus efficient planning. In Blue Care, performing Primary Care Plus in independent centres is expected to solve the inefficient use of consultation hours. Some literature showed that care in outreach clinics leads to shorter waiting times, fewer follow-up visits and a higher level of satisfaction with clinical processes. However, care in outreach clinics can also lead to increased healthcare costs [10,12,31], and additional overhead costs [32].

The fear of losing the close relationship between general practitioners and medical specialists in Primary Care Plus centres is not irrelevant according to the literature since the direct communication between general practitioners and medical specialists seemed to have positive influence on the perceived quality of care and health outcomes compared to a system where medical specialists work in a primary care setting without direct contact with general practitioners [15,32,33]. As a replacement, the steering committee opted for joint consultation models [16,17,19]. The question is whether these models are enough to fill the gap of losing direct communication between general practitioners and medical specialists. Future research should determine whether these concerns are justified.

This study showed that general practitioners experienced a problem with referring patients to the correct place for care, i.e. Primary Care Plus or the hospital. A group of patients was referred to hospital care while they belonged in Primary Care Plus and vice versa. Essential for achieving effective and efficient substitution is to select the appropriate patient population that requires low complex care without needing the facilities of the hospital. Without this proper selection, Primary Care Plus can become an intermediate station between Primary Care Plus and hospital care. General practitioners should thus be able to deliberate with medical specialists about referral uncertainties. Future research is needed to determine whether a learning effect for general practitioners occurs. The suggestion of the steering committee to select patients according to the International Classification of Primary Care coding system contrasts with the idea of the general practitioner as a gatekeeper as general practitioners lose their autonomy in referring patients to specialist care. It is therefore questionable whether this is the correct solution for this problem.

The specialty of cardiology seemed to not be suitable for Primary Care Plus in its current application. Furthermore, cardiologists and orthopaedics needed more diagnostic facilities than currently available in Primary Care Plus. The current approach of Primary Care Plus with minimal diagnostic facilities makes it questionable if all medical specialties are suitable. Future research should confirm whether the assumption by neurologists and internists that they requested less diagnostic tests in Primary Care Plus is true. The joint consultation model showed that patients underwent fewer diagnostic tests in the intervention group compared to the patients who received hospital care [16,17].

### Strengths and limitations

In contrast with other studies on shifting health care or shared care between primary and hospital care, the scope of this study was on strengthening the cooperation and substitution in a population management setting, while many other studies focused only on specific disease management programmes [34,35,36,37]. This makes the social and scientific relevance stronger.

This study used a qualitative responsive approach with different resources and a member check which resulted in increased reliability and internal validity [18]. However, this study provided no insight yet into referral patterns and the Triple Aim outcomes for Primary Care Plus. In addition, one should keep in mind that this region has some specific characteristics (only one primary care organisation, one (academic) hospital, a long tradition of collaboration). This means that some region specific results cannot be extrapolated to other regions in the Netherlands and beyond.

### Future research

Therefore, in the coming years, the ‘Academic Collaborative Centre for Sustainable Care’ of Maastricht University will focus not only on the Triple Aim outcomes in Blue Care but will also monitor and evaluate other initiatives in the South of Limburg using different kinds of Primary Care Plus interventions. Effort will be placed in the development of implementation protocols for Primary Care Plus, dependent on the Primary Care Plus model and the context of the region.

## Conclusion

The findings of this study on the barriers, facilitators and necessary preconditions of Primary Care Plus resulted in relevant input for changing the design of substituting hospital care with primary care in the context of ‘Blue Care’. With an observational study design using mixed methods, we will monitor and evaluate whether this new approach of placing medical specialists in Primary Care Plus centres outside the venue of the hospital will result in Triple Aim objectives. Other initiatives in the South of Limburg using different kinds of Primary Care Plus interventions will be subject to research in the near future as well. Best practices will be developed by comparing all these different initiatives with the intention of making health care sustainable for future generations.

## Competing Interests

The authors declare that they have no competing interests.

## References

[1] OECD, Health policies data (2014). http://www.oecd.org/els/health-systems/health-spending-starts-to-rise-but-remains-weak-in-europe.htm.

[2] Ewijk C, Horst van der A, Besseling P (2013). The future of health care.

[3] Rijksoverheid (2012). [Dutch Government]. Naar beter betaalbare zorg. Rapport Taskforce
Beheersing Zorguitgaven. [To more affordable care. Report Taskforce Control
Healthcare Expenditures.].

[4] Actiz, CSO, GGD, GGZNederland, KNMG, LHV, LVG, NFU, NPCF, NVZ, Orde van Medisch Specialisten, VGN, V&VN, Zorgverzekeraars Nederland (2012). De agenda voor de zorg. Aanbod aan politiek en samenleving van
het zorgveld. [The agenda for care. An offer to politics and society of the
healthcare sector.].

[5] Ministerie van Volksgezondheid, Welzijn en Sport (2013). Onderhandelingsresultaten Schippers met ziekenhuizen, medisch
specialisten, zelfstandige behandelcentra, GGZ en huisartsen. [Negotiation
results of Minister Schippers with hospitals, medical specialists,
independent treatment centres, mental health care and GPs.].

[6] Schulpen G, Meerlo R, Uden van C, Dekkers T, Hees van A, TBlauwe Zorg (2012). Regio-experiment voor duurzame zorg in Maastricht en Heuvelland.
[Blue Care. Region experiment for sustainable health care in Maastricht and
Heuvelland.].

[7] Berwick DM, Nolan TW, Whittington J (2008). The triple aim: care, health, and cost. Health Affairs.

[8] Bisognano M, Charles K (2012). Leadership for the triple aim. Three-pronged framework helps
executives lead quality initiatives. Healthcare Execution.

[9] Bisognano M, Kenney C (2012). Pursuing the triple aim: seven innovators show the way to better
care, better health, and lower costs.

[10] Bailey JJ, Black ME, Wilkin D (1994). Specialist outreach clinics in general practice. British Medical Journal.

[11] Bond M, Bowling A, Abery A, McClay M, Dickinson E (2000). Evaluation of outreach clinics held by specialists in general
practice in England. Journal of Epidemiology & Community Health.

[12] Bowling A, Bond M (2001). A national evaluation of specialists’ clinics in primary
care settings. British Journal of General Practice.

[13] Bowling A, Stramer K, Dickinson E, Windsor J, Bond M (1997). Evaluation of specialists’ outreach clinics in general
practice in England: process and acceptability to patients, specialists, and
general practitioners. Journal of Epidemiology & Community Health.

[14] Gosden T, Black M, Mead N, Leese B (1997). The efficiency of specialist outreach clinics in general
practice: is further evaluation needed?. Journal of Health Services Research & Policy.

[15] Gruen R, Weeramanthri T, Knight S, Bailie R (2006). Specialist outreach clinics in primary care and rural hospital
settings (Cochrane Review). Community Eye Health Journal.

[16] Vierhout W, Knottnerus J, Crebolder H, Wesselingh-Megens A, Beusmans G, Ooij van A (1995). Effectiveness of joint consultation sessions of general
practitioners and orthopaedic surgeons for locomotor-system
disorders. The Lancet.

[17] Vlek J, Vierhout W, Knottnerus J, Schmitz J, Winter J, Wesselingh-Megens A (2003). A randomised controlled trial of joint consultations with general
practitioners and cardiologists in primary care. British Journal of General Practice.

[18] Hulscher M, Laurant M, Grol R (2003). Process evaluation on quality improvement
interventions. Quality and Safety in Health Care.

[19] Schulpen G, Vierhout W, Heijde van der R, Landewe R, Winkens R, Linden van der S (2003). Joint consultation of general practitioner and rheumatologist:
does it matter?. Annals of the rheumatic diseases.

[20] Hsieh H, Shannon SE (2005). Three approaches to qualitative content analysis. Qualitative Health Research.

[21] Lincoln Y, Guba E (1985). Naturalistic inquiry.

[22] Borst-Eilers E, Sorgdrager W (1998). Wet medisch-wetenschappelijk onderzoek met mensen. [Dutch Medical
Research (Human Subjects) Act.].

[23] Sorgdrager W (1997). Wet van 22 mei 1997, houdende nieuwe regels omtrent de
economische mededinging (Mededingingswet). [Act of 22 May 1997, laying down
new rules on economic competition (Competition act).].

[24] Schulpen G (2003). The joint consultation of general practitioners and
rheumatologists. Research Institute for Extramural and Transmural Care.

[25] Vrijhoef H, Spreeuwenberg C, Eijkelberg I, Wolffenbuttel B, Merode van G (2001). Adoption of disease management model for diabetes in region of
Maastricht. British Medical Journal.

[26] Hardy B, Mur-Veemanu I, Steenbergen M, Wistow G (1999). Inter-agency services in England and The Netherlands. A
comparative study of integrated care development and
delivery. Health Policy.

[27] Koelen MA, Vaandrager L, Wagemakers A (2012). The healthy alliances (HALL) framework: prerequisites for
success. Family Practice.

[28] Gruen RL, Weeramanthri TS, Bailie RS (2002). Outreach and improved access to specialist services for
indigenous people in remote Australia: the requirements for
sustainability. Journal of Epidemiology & Community Health.

[29] Gillam S, Ball M, Prasad M, Dunne H, Cohen S, Vafidis G (1995). Investigation of benefits and costs of an ophthalmic outreach
clinic in general practice. British Journal of General Practice.

[30] Helliwell P (1996). Comparison of a community clinic with a hospital out-patient
clinic in rheumatology. Rheumatology.

[31] Ayshford C, Johnson A, Chitnis J (2001). What is the value of ENT specialist outreach
clinics?. The Journal of Laryngology and Otology.

[32] Powell J (2002). Systematic review of outreach clinics in primary care in the
UK. Journal of Health Services Research & Policy.

[33] Gruen RL, Weeramanthri TS, Knight SE, Bailie RS (2004). Specialist outreach clinics in primary care and rural hospital
settings. Cochrane Database of Systematic Reviews.

[34] Drummond N, Abdalla M, Buckingham J, Beattie J, Lindsay T, Osman L (1994). Integrated care for asthma: a clinical, social, and economic
evaluation. British Medical Journal.

[35] Jolly K, Bradley F, Sharp S, Smith H, Thompson S, Kinmonth A (1999). Randomised controlled trial of follow up care in general practice
of patients with myocardial infarction and angina: final results of the
Southampton heart integrated care project (SHIP). British Medical Journal.

[36] Rothman AA, Wagner EH (2003). Chronic illness management: what is the role of primary
care?. Annals of Internal Medicine.

[37] Smith SM, Allwright S, O’Dowd T (2007). Effectiveness of shared care across the interface between primary
and specialty care in chronic disease management. Cochrane Database of Systematic Reviews.

